# Online trend estimation and detection of trend deviations in sub-sewershed time series of SARS-CoV-2 RNA measured in wastewater

**DOI:** 10.1038/s41598-024-56175-2

**Published:** 2024-03-06

**Authors:** Katherine B. Ensor, Julia C. Schedler, Thomas Sun, Rebecca Schneider, Anthony Mulenga, Jingjing Wu, Lauren B. Stadler, Loren Hopkins

**Affiliations:** 1 Department of Statistics, Rice University, 6100 Main St., Houston, TX 77005 USA; 2 Department of Civil and Environment Engineering, Rice University, 6100 Main St, Houston, TX 77005 USA; 3 Houston Health Department, 8000 N. Stadium Dr., Houston, TX 77054 USA; 4 Houston Health Department and Department of Statistics, Rice University, 6100 Main St., Houston, TX 77005 USA

**Keywords:** Statistics, Epidemiology, Statistics, Epidemiology

## Abstract

Wastewater surveillance has proven a cost-effective key public health tool to understand a wide range of community health diseases and has been a strong source of information on community levels and spread for health departments throughout the SARS- CoV-2 pandemic. Studies spanning the globe demonstrate the strong association between virus levels observed in wastewater and quality clinical case information of the population served by the sewershed. Few of these studies incorporate the temporal dependence present in sampling over time, which can lead to estimation issues which in turn impact conclusions. We contribute to the literature for this important public health science by putting forward time series methods coupled with statistical process control that (1) capture the evolving trend of a disease in the population; (2) separate the uncertainty in the population disease trend from the uncertainty due to sampling and measurement; and (3) support comparison of sub-sewershed population disease dynamics with those of the population represented by the larger downstream treatment plant. Our statistical methods incorporate the fact that measurements are over time, ensuring correct statistical conclusions. We provide a retrospective example of how sub-sewersheds virus levels compare to the upstream wastewater treatment plant virus levels. An on-line algorithm supports real-time statistical assessment of deviations of virus level in a population represented by a sub-sewershed to the virus level in the corresponding larger downstream wastewater treatment plant. This information supports public health decisions by spotlighting segments of the population where outbreaks may be occurring.

## Introduction

Wastewater-based epidemiology (WBE) is an approach to population disease monitoring which collects samples from a community’s wastewater system and evaluates those samples for the presence and abundance of a given disease-causing pathogen. WBE is a cost-effective and fast way to survey the transmission of disease in populations, and it has been widely applied for the monitoring of viral pathogens, including SARS-CoV-2^1,2^. Studies demonstrate that wastewater-based epidemiology can be used as an early warning for a potential increase in cases of a disease, in this case SARS CoV-2^3^. Other studies have examined different targets such as influenza and RSV^4^. Comprehensive programs demonstrate the value of WBE to public health intervention^5^. A recent systematic overview of the global application of WBE for the detection of SARS-CoV-2 addresses issues from sampling and lab methodologies to statistical and mathematical methods for assessment and prediction of SARS-CoV-2 cases in monitored populations^6^. An extensive list of estimated Pearson and Spearman correlations between virus levels in wastewater and observed cases that span the globe is provided. The association between observed virus levels in wastewater and prevalence of SARS-CoV-2 in the monitored population is clear. We contribute to the literature for this important public health science by putting forward time series methods coupled with statistical process control that (1) capture the evolving trend of a disease in the population; (2) separate the uncertainty in the population disease trend from the uncertainty due to sampling and measurement; and (3) support comparison of sub-sewershed population disease dynamics with those of the population represented by the larger downstream treatment plant. Our methods incorporate the fact that measurements are over time.

Viral concentrations in wastewater sampled over time from a given location form a time series. The relationship between a time series of observed virus concentrations in wastewater and the number of positive tests for the same population has been explored using Pearson correlation^7,8^. The Pearson correlation analysis does not account for temporal dependence likely to be present in the data. If temporal dependence is ignored, a concern is that findings of a statistically significant relationship between wastewater viral concentration and population case counts can become insignificant. Temporal correlation, estimated by the autocorrelation (ACF) and partial autocorrelation (PACF), of weekly averaged N1 and N2 WWTP concentrations is statistically significant^9^. Many study conclusions, however, report on p-values computed using Pearson correlation and other statistical methods which assume there is no temporal correlation. The challenges of analyzing time series data are important to consider^10^. Time series methods have been employed in the WBE literature, including copula time series models^11^ and gradient boosting trees with a time lag^12^. Several studies have applied an Autoregressive Integrated Moving Average (ARIMA) time series model to forecast covid-19 cases using wastewater viral concentrations^11,13,14^. We contribute to this literature with a new time series approach.

The time series methods mentioned in the literature account for temporal correlation in the observed series of wastewater concentrations, but only estimate a single variance term. In other words, variability specifically due to the measurements taken via sampling the wastewater and processing the samples in the lab is not separated from variability inherent to the trend. One study comparing lagoons to upstream pump stations revealed that the upstream sampling sites yielded better measurements than the lagoons^15^. In contrast, another study comparing viral concentrations measurements taken from wastewater treatment plants (WWTPs), pump stations, and manholes found no difference in the mean concentrations of smaller upstream community sewershed areas and their respective treatment centers^16^. Population size may affect the variability of observations for a particular location^17^. Population-normalization of the viral concentrations adjusted for wastewater flow rates is a way to address population size as a source of variability. Different systems and different locations within the same system may give different information, so the ability to compare trends across these locations is essential for extracting the most information possible from routine and as-needed sampling. Some methods need large amounts of data before models can provide actionable answers from a public health perspective^15^. We develop methods that can be confidently applied to shorter time series, which may only have a small amount of data.

This work explores a combination of two statistical techniques: one to quantify the impact of sampling and measurement error and one to determine whether samples from a new location are deviating from routine measurements. First, we model the wastewater measurements using a dynamic non-linear state-space time series model. This modelling framework allows for both online and retrospective estimation of wastewater trends accompanied with confidence bands to capture the precision of the estimates^18^. The method is valid with small sample sizes, but increases in precision with the sample size. These estimates give broad insight into whether sub-sewershed time series provide different public health information from large, centralized WWTP time series. Although not the focus of this manuscript, the time series methodology put forward supports near-term forecasts with uncertainty quantification. Second, we utilize tools from statistical process control (SPC), namely exponentially weighted moving average (EWMA) control charts, to monitor whether lift station measurements deviate significantly from the trend estimate for the larger WWTP. Our combined statistical methodology will provide an assessment for a single observation from a sub-sewershed, allowing for immediate online detection of community-specific spikes in SARS-CoV-2.

## Results

### Data description

**Figure 1:**
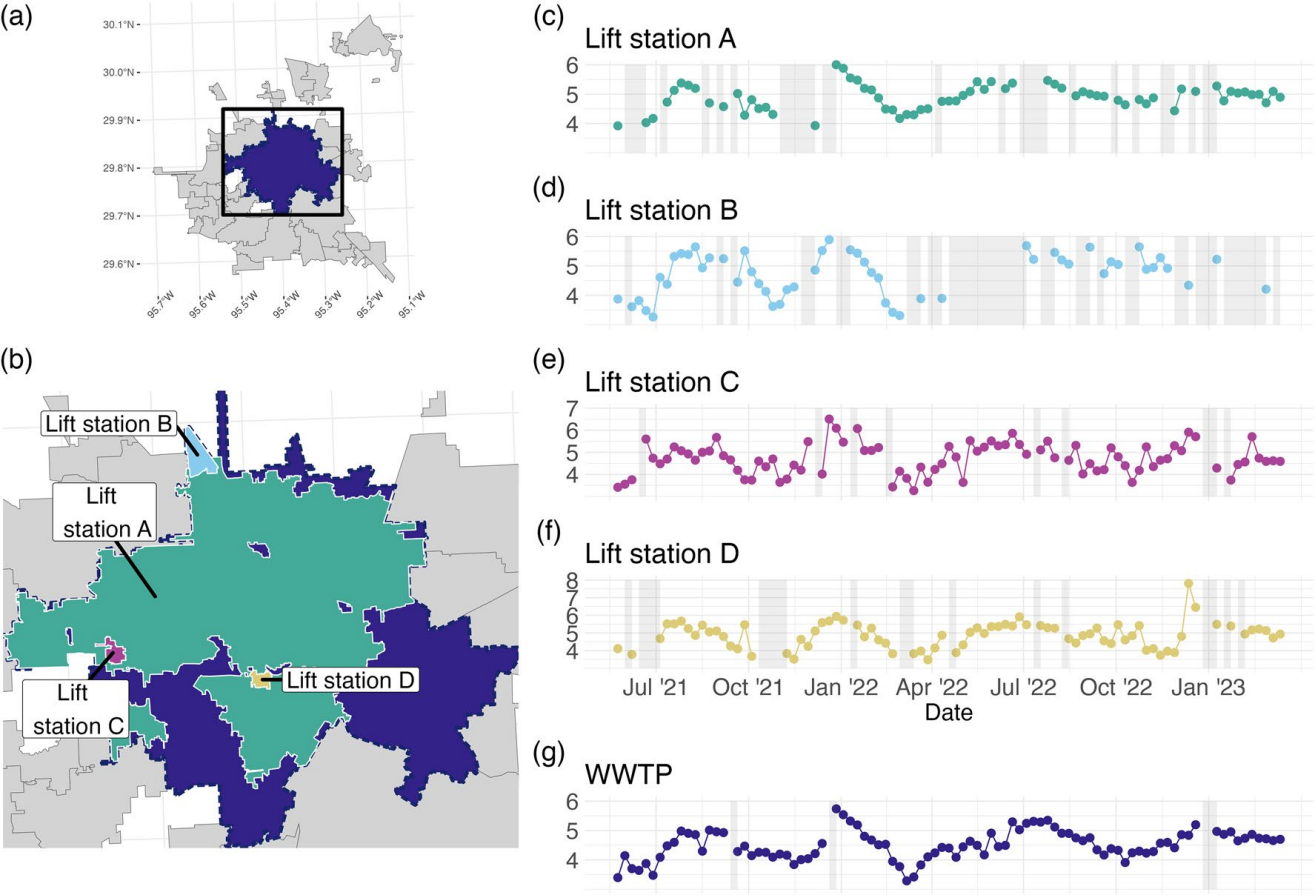
(**a**) The WWTP catchment areas for the City of Houston, with the WWTP of focus shaded. The box shows the extent of (**b**), the map showing the 4 lift stations considered in the analysis. (**c**–**g**) Plot the time series of Log10 Copies/L for the WWTP and the 4 lift station facilities, referred to as Lift Station A–D, with periods of missing values indicated by grey rectangles.

The City of Houston has 39 WWTPs, serving populations from approximately 500,000 to 600 individuals. Within the larger WWTPs, there are a number of lift station (LS) facilities where wastewater can be sampled and may serve to refine the geographic resolution provided by wastewater analysis. This work focuses on the largest WWTP that serves a population of roughly 551,150 people. Wastewater was sampled from May 24, 2021 through March 13, 2023 for four lift stations (see Fig. 1) which are geographically contained within the large WWTP catchment area.

Data on wastewater analysis results for the lift stations and the WWTP was updated on a weekly basis. For each weekly sample, we quantified SARS-CoV-2 N1 and N2 gene copies per liter of wastewater, as described in a previous study concerning WBE in the City of Houston^19^. We average the N1 and N2 concentrations to simplify our analysis and focus on the comparison between the WWTP and LS time series. All measurements taken in a given week were aligned to the corresponding Monday of that week. Wastewater viral concentration data was received in units of copies per liter and was subsequently log transformed in base 10 ($\log _{10}$). Any measurements below the level of detection (LOD) were labeled as missing values. Table 1 contains the names of the 5 series considered and summary statistics for each series. Figure 1a and b are maps of the WWTP and LS catchments for each of the series, and Fig. 1c–f plot the time series of observed values for all 5 series on the $\log _{10}$ scale.

**Table 1:** Name, size of population, and summary statistics Log10 of average of replicate RNA N1 and N2 copies/L for each wastewater treatment plant (WWTP) or lift station (LS) considered. The study period spanned 95 weeks.

Name	Population	Mean	St. Dev.	Min	Max	Missing
WWTP	551150	4.51	0.50	3.29	5.74	4
Lift Station A	373937	4.89	0.43	3.92	6.00	28
Lift Station B	4849	4.72	0.73	3.26	5.89	42
Lift Station C	2442	4.72	0.70	3.26	6.51	11
Lift Station D	1724	4.88	0.74	3.48	7.81	18

### Estimation of trends in wastewater time series

**Figure 2:**
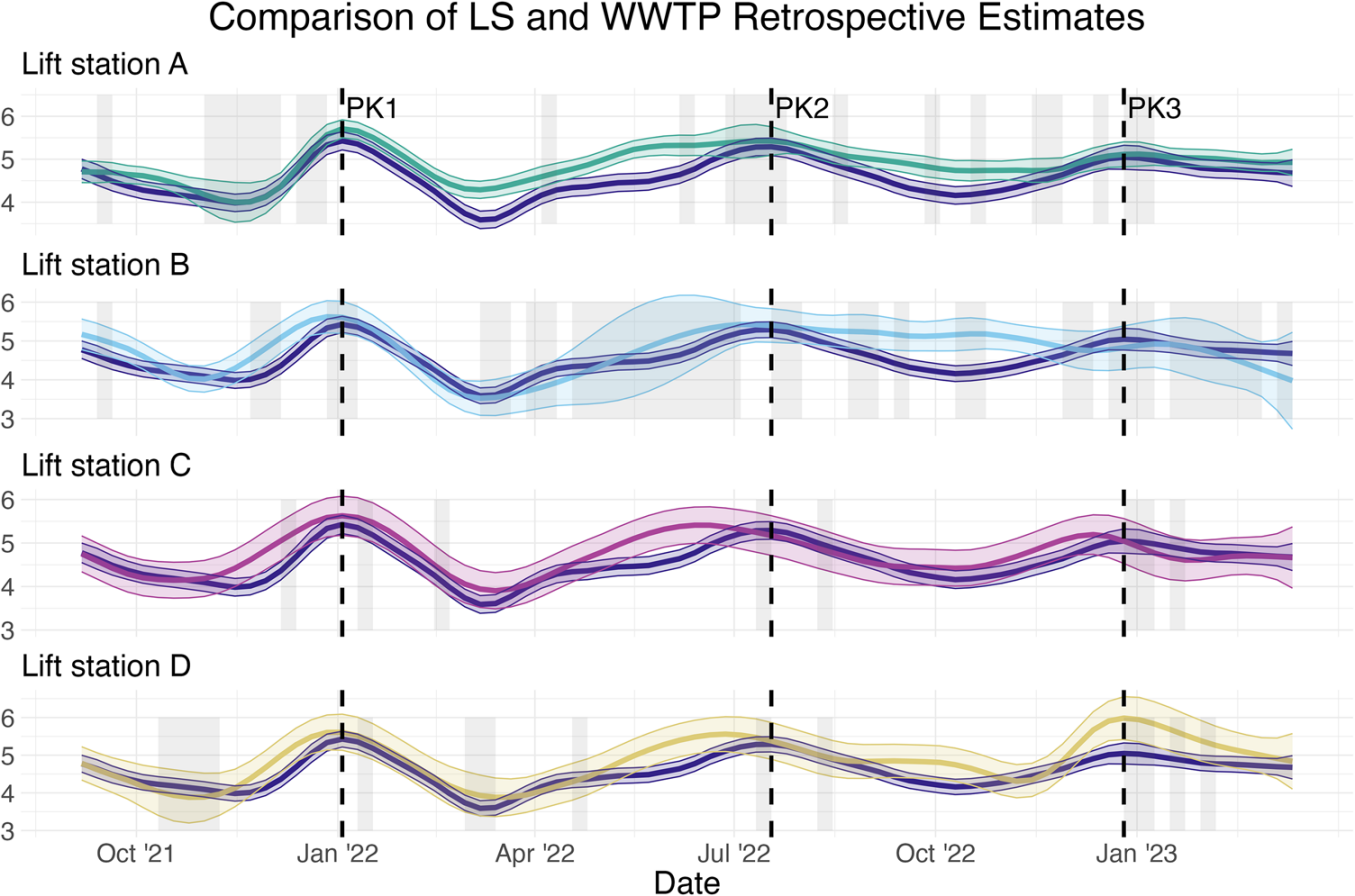
Retrospective estimates of the viral concentration trend with uncertainty quantification for the WWTP (the blue curve repeated each time) and each LS series, using all available information. The vertical axis is $\log$10 copies/liter. The shaded grey rectangles correspond to periods of missing data and the dotted lines correspond to the peaks of three surges. Note that the time series model is still able to provide estimates of the trend during periods of missing data, though with greater uncertainty. Compared to Fig. 1, the start date of this trend plot is later, since the first 10 weeks of data are used to initialize the model.

The statistical model is a time series model that gives estimates of the true viral concentration as well of estimates of the uncertainty at each time point. Further, these estimates can use either all data from the study period (retrospective) or just until the current time point (online). The model is fit separately to each wastewater time series. The retrospective trend estimates depicted in Fig. 2 indicates three peaks in the estimated population viral dynamics for the population served by the WWTP, with maximums that occur on January 3 and July 18, 2022, and January 9, 2023. We will refer to these peaks as PK1, PK2 and PK3, respectively. The retrospective review illustrates there are instances where the lift stations provided early information with respect to increasing or decreasing viral trends in the population measured. A separation in the confidence intervals for each series indicates a statistically significant difference in the estimated trend for the respective series, namely the trend estimated for the WWTP and each of the lift stations. This assessment of statistical significance takes into account the measurement and sampling error for each location.

We see in the retrospective review that three of the four lift stations, namely, Lift stations B, C and D, exhibit a comparable trend as that estimated from the WWTP, with a few deviations (see Fig. 2). Lift stations B, C, and D exhibited more variation in their trend estimates as evidenced by the width of the respective confidence intervals, than that of the WWTP and Lift Station A. There are several missing values in the Lift station B series as highlighted by the light grey bars in Fig. 2. The estimated level for Lift station B does not show an increase in uncertainty between PK2 and PK3 due to the fact that the observed levels during this time were relatively consistent (see Fig. 1). Lift station B statistically separates from the WWTP trend by remaining at high levels between PK1 and PK2. Lift station C indicates an early signal leading up to PK2. Lift station D registers a statistically higher trend during PK3. Lift station A was unique amongst the four lift stations, in that its estimated trend separated from the estimated WWTP trend following PK1 and remained higher until PK2. Lift station A failed to drop as low between PK2 and PK3. The trend estimates for the WWTP and Lift station A were not significantly different during PK3.

The hierarchical trend estimation framework can separate variability associated with the trend from the “noise”, or lab and sampling error. These separated estimates are summarized in Table 2. We see from this table that the measurement and sampling variation are highest for Lift station C and D, and also elevated for Lift station B. Since we expect the lab variability to be approximately constant across all measurements, the extra variation is most likely due to the lift station wastewater sample containing highly variable levels of SARS-CoV-2 due to the small population that it serves, and possibly related to wastewater flow rate for the location. The sampling variation is approximately equivalent for Lift station A as it is for the WWTP. The state dynamics for each location exhibit similar variability, with slightly elevated variation for Lift station D.

**Table 2:** Estimates of inherent variability, $\sigma _w$ (state) and measurement variability $\sigma _v$ (observation, lab and sampling variability) for each series.

Name	Sampling and lab variability	Trend variability	Population
WWTP	0.0372	0.0130	551,150
Lift station A	0.0350	0.0130	373,937
Lift station B	0.1374	0.0134	4849
Lift station C	0.2798	0.0105	2442
Lift station D	0.2810	0.0175	1724

### Detection of deviations between two wastewater time series

**Figure 3:**
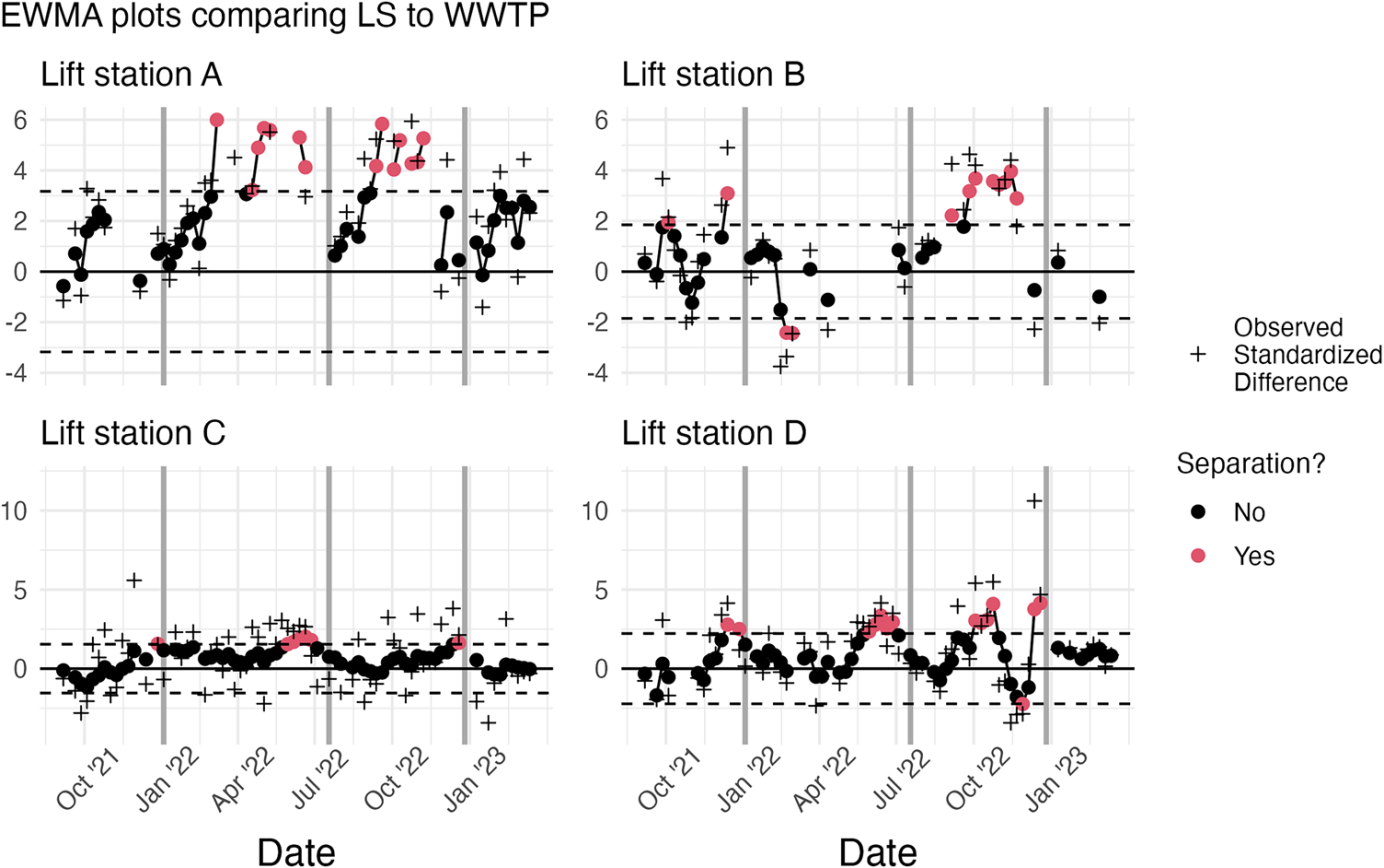
The EWMA chart for the observed values at each lift station compared to the WWTP online estimate. The solid dots represent the exponentially weighted standardized difference while the plus signs represent the actual standardized difference. Observations which correspond to a structural break, or exponentially weighted values beyond the dotted control limits, are colored red. The dark grey vertical lines are the approximate dates of the peaks of different surges.

The Exponentially Weighted Moving Average (EWMA) control chart is a Statistical Process Control (SPC) methodology which can detect when two time series are separating. The EWMA method requires 10 temporal observations from a large, routinely monitored WWTP and at least one point from a second location to determine whether the measurement(s) from the second location are consistent with the first. Figure 3 visualizes the control charts which allow for the detection of lift station deviations from WWTP trends. These charts are the result of Algorithm 2 applied to each lift station and the WWTP. The information is consistent with the retrospective study in that Lift station B, C, and D, all demonstrate minor perturbations from the trend estimated for the WWTP. Further, Lift station A clearly demonstrates a strong and consistent deviation from the WWTP estimated trend, between PK1 and PK2, and then again between PK2 and PK3. In Fig. 3 we also include the observed standardized difference between the two measurements. You will note that the differences may be large, but they are not always statistically significant based on the EWMA control chart. The control chart is used to identify a level shift in the trend, and not specific outlying events. Based on the EWMA control chart, statistically significant level shifts occurred at the red highlighted temporal locations.

## Discussion

The objective of this paper is to bring forward statistical methods which respect the temporal dependence of the data, can separate measurement and sampling error from variation in the population disease dynamics, and provide insight into whether different information is gleaned from upstream sampling sites. In addition to separating sources of variation, the hierarchical time series approach captures the dynamic trend in population viral dynamics from each wastewater series. This time series model is simple to implement both in a retrospective, and real-time mode, and naturally adapts to the nonlinear dynamics in the population viral trend. The EWMA control charts provide a framework for identifying when measurements derived from sub-sewersheds deviate from trends in a larger, centralized WWTP, accounting for the inherent sampling and measurement variation in both.

The ability to gain insight into whether a specific measured location differs from the main population trend, or is “out of control”, with just a few observations for a new location is a useful in public health decision making for several reasons. The literature has increasingly explored sampling of upstream sites, such as schools^4,20,21^ or buildings^22^, as a way to gain insight into community spread. Once a new sampling location is identified, statistical methods described here provide statistically sound preliminary information, with sensitivity improving as more information becomes available. Vector autoregressive models (VAR), have been applied successfully with large amounts of data to predict case counts using wastewater^23^. Additionally, a state space modeling formulation could be explored for epidemiological SEIR models^24^.

Based on the control charts in Fig. 3, the only lift station for our system within the large wastewater catchment area of the WTTP whose trend consistently deviated from that of the WWTP was Lift station A. The measurement and sampling uncertainty for Lift station A was on par with that of the WWTP. This lift station serves 373,937 people whereas the WWTP serves 551,150 people. In other words, Lift station A serves 68% of the people in the large catchment area. Regular monitoring of Lift station A in addition to the WWTP is warranted based on this study. The Lift station A state estimate of viral load and its uncertainty, indicates that for the 68% of the population served by Lift station A, the viral load did not decrease as substantially as that of the WWTP between PK1 and PK2, and also between PK2 and PK3. The methods described here provide rapid answers when public health decisions are to be made based on samples from smaller communities within a larger system. The control charts are illustrated for 95 weeks of data in total, however, the leftmost dots on the plot required only 10 weeks of data from a regularly monitored site and just one observation from a new sampling site. The control charts are used in an on-line fashion.

For the lift stations serving smaller populations, namely Lift station B, C and D, we see evidence of early signals through each COVID-19 peak. However, the measurement and sampling uncertainty with these smaller lift stations was substantially higher. Although routine monitoring may be prohibitively expensive, monitoring through times of high concern to public health may be warranted. Rapid statistically valid conclusions are supported by the coupled times series and EWMA approach we have articulated.

An attractive feature of our modeling approach is the opportunity to separate the variation in the trend of the community virus levels from the measurement and sampling variation. In this comparison, and assuming a consistent measurement or lab variability across all samples, we find that the sampling variation for the smaller lift stations is much greater than that for the WWTP and the large Lift station A.

## Methods

### Hierarchical time series model for trend estimation

When time series data are collected, the goal is often to estimate a trend, that is, whether the “typical values” are changing in time. For example, Fig. 1c–g show the times series of viral concentration of SARS-CoV-2. A quick visual inspection indicates that these values are changing in time, and even seem to exhibit similar behavior that may be predictable with a well-chosen statistical model. Such a model should be able to separate out the “noise”, or observation/measurement error, in these observations from the “signal”, or trend. An additional constraint when modeling time series data is the presence of temporal correlation structure, i.e. the values are not independent, so models which assume independence can lead to misleading forecasts and/or conclusions about which variables are important in modeling a time series. The desired model will separate sources of variability for both the trend and the observation as well as account for temporal correlation.

The state space modeling framework can accommodate both these needs. A state space model represents a time series in two levels: an unobserved trend which encodes temporal dependence structure and a noisy observed time series. In other words, it is a hierarchical model which is able to separate sources of variability as desired. In the time series literature, the levels of this model are called the state equation and the observation equation. Equations (1) and (2) display the state space model used for each series in this particular study:

1 $$\begin{aligned} \text {Observation equation: }&y_{t} = \mu _t + v_{t} \end{aligned}$$

2 $$\begin{aligned} \text {State equation: }&(\mu _t - \mu _{t-1}) = (\mu _{t-1} - \mu _{t-2}) + w_t. \end{aligned}$$

3 $$\begin{aligned} \text {Initial condition: }&\mu _0 \sim N(c_0, m_0). \end{aligned}$$

The error terms $v_t$ and $w_t$ are independent and normally distributed with mean zero, and variances denoted by $\sigma ^2_v$ for the observation error and $\sigma ^2_w$ for the state error.

The observation model of Eq. (1) represents the model fit to the concentration of SARS-CoV-2 RNA in wastewater measured by the lab. The observation model is the underlying state $\mu _t$ plus a variance term $\sigma ^2_v$ corresponding to the inherent measurement and sampling error. The state model in Eq. (2) represents the true state of the viral trend derived from the measured concentration of SARS-CoV-2 RNA, for the sampled region. The noise term associated with the state equation, $\sigma _w^2$, represents the natural variability in the viral concentration in the population as measured by wastewater.

Within this framework, the state variable serves as the core component of the model, characterizing the underlying system’s behavior and dynamics, in other words the trend of the virus concentration. Note the temporal structure encoded by Eq. (2): the right hand side concerns the previous two time points, while the left hand side concerns the current and past time point. Equation (2) encompasses a statistical framework that employs the concept of first difference applied twice. The first difference operation captures the change in the state variable over successive time periods, and by applying this operation twice, we gain insights into the acceleration or curvature of the trend. This choice of structure for the state equation is chosen to capture the temporal dependence of the SARS-CoV-2 RNA concentration and has been used previously though, not in a state-space framework^5^. For additional details about the state-space modeling framework and its relations to smoothing splines, see^18^.

Once the structure of the model is chosen, the model can be fit to the data with three goals in mind: retrospective estimates of the trend using all available data, online estimates of the trend using only past data up to a given time point, and one-step-ahead forecasts of the next time point. In the time series literature, the retrospective and online estimates are referred to as smoothers and filters, respectively. We focus on retrospective and online estimates for this paper, but provide steps for obtaining the one-step-ahead forecasts in the supplemental materials.

To estimate the online and retrospective trends, four parameters are themselves estimated: the initial state mean and variance, the variance of the measurement and sampling error ($\sigma _v^2$), and the variance of the trend ($\sigma _w^2$). Estimates are obtained through maximum likelihood estimation which is computationally fast due to use of the Kalman Filter for updating linear Gaussian systems. For the online estimates, a rolling estimation structure is used, meaning the parameters are re-estimated with each new time point. Estimation is implemented using the KFAS package in R^25^, which can easily handle missing data. Once an estimate of the model is obtained, a step to check that the model fits the data is required. For the present model, an autocorrelation plot of the model’s residuals can be checked for autocorrelation. If no correlation is present in the residuals, the model can be considered a good fit. Additional details of estimation and model fit checks as well as all code used for the analysis are available in the supplemental materials.

**Figure 4:**
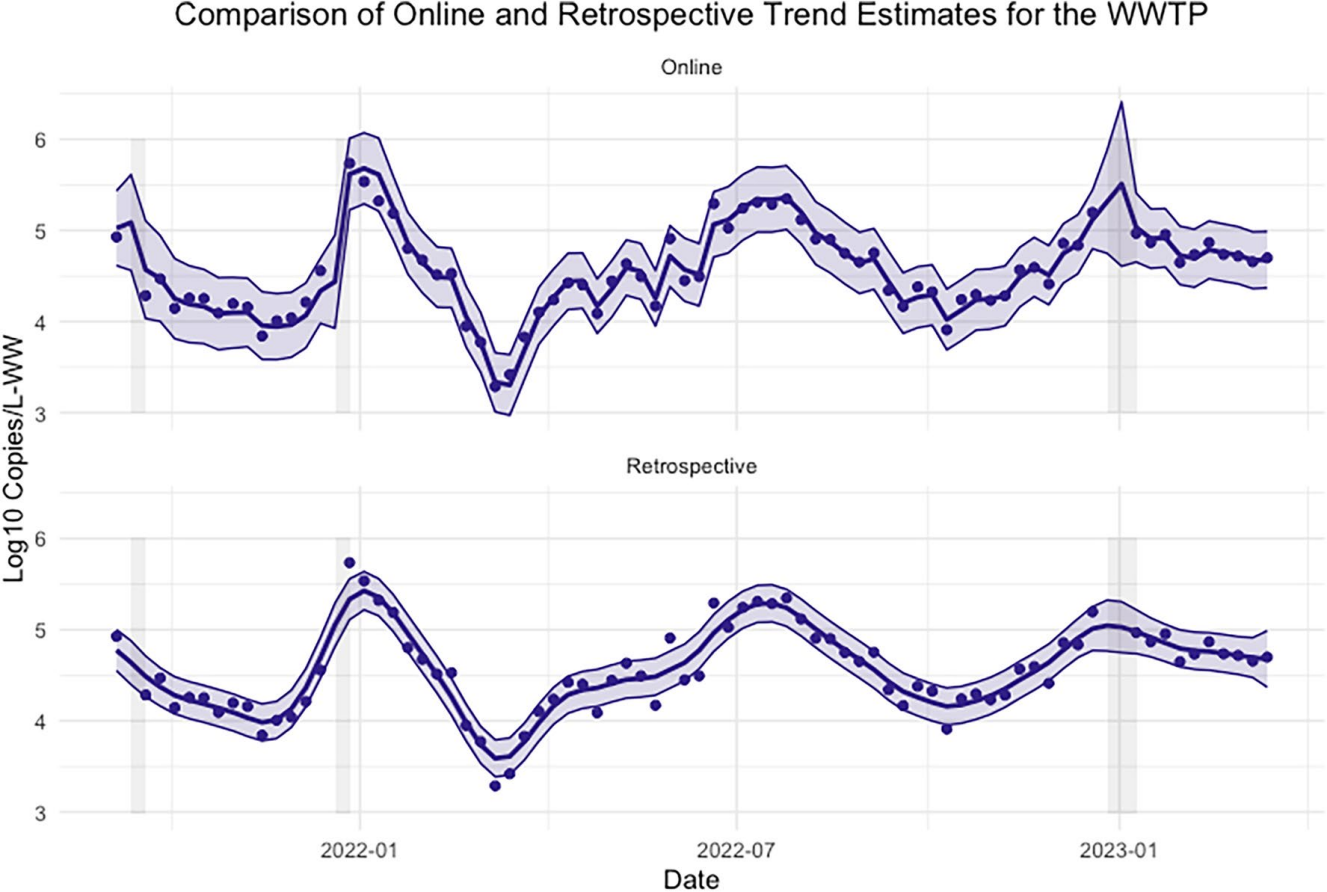
Retrospective and online estimates of the viral concentration trend with uncertainty quantification for the large WWTP. The vertical axis is $\log$10 copies/liter. The shaded grey rectangles correspond to periods of missing data. Note that the online trend estimates are “noisier” and have wider uncertainty bands than the retrospective trend estimates.

The inputs, outputs, and process of fitting the spline state space model of Eqs. (1) and (2) are summarized in Algorithm 1.Visualizations of the retrospective and online estimates along with the data are provided in Figure 4.

**Algorithm 1:**
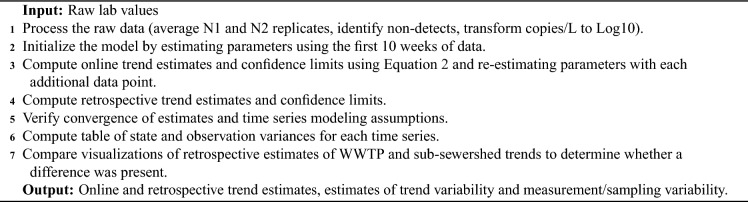
Variability-separating trend estimation.

### Detection of trend deviations

Recall the goal of determining whether sub-sewershed measurements give different information than the routinely monitored centralized WWTP measurements. Using all available data, the retrospective estimates from the model fit using Eqs. (1) and (2). These estimates, visualized in Fig. 2, show some periods of separation, indicating that the sub-sewershed measurements do indeed give different information. However, if the goal is to extract actionable information from the data, the online estimates, which only use data up to the current time point, should be used. While the retrospective estimates show clear separation, the online estimates are noisier, so detecting when the sub-sewersheds may be deviating from the WWTP’s trend requires more than a visual comparison of the two series. In addition, sub-sewersheds may not be sampled frequently enough to support the model described in the “Methods” section, so a method which can be used with at least one sub-sewershed observation is ideal.

The statistical process control (SPC) literature provides a framework for iterative improvement of a decision-making process based on time series data. Some examples of the traditional applications of SPC include ensuring a given percentage of on-time deliveries to a client, speed and consistency of service quality in a bank, and loading passengers onto an airplane^26^. In short, SPC provides a framework for identifying when a time series of interest is “out of control” so that steps can be taken to bring that series back “in control”. Although the ability to bring disease burden in a community back “in control” is limited in WBE compared to traditional applications, ideas from SPC can be borrowed to improve the actionability of the information contained in wastewater time series.

For this paper, the time series of interest is the difference between the sub-sewershed and the WWTP. If we simply subtract the observed values for each series, the resulting difference will contain the “noise”, or measurement and sampling error. Instead, we use the online estimate of the trend for the WWTP obtained from Eqs. (1) and (2), which can be assumed to be free of observation error. Since the online estimate of the trend requires 10 weeks of data to be initialized, we use the observed (unmodeled) value(s) from the sub-sewershed directly.

Using the previous notation, the standardized difference at time point *t*, for lift station $i=1,\ldots ,4$ is given by:

4 $$\begin{aligned} d_{i,t} = \frac{y_{i,t} - \hat{\mu }_t}{\tilde{\sigma }_d}, \end{aligned}$$

where $\tilde{\sigma }_d^2 = \textrm{Var}(y_{i,t} - \hat{\mu }_t).$ This variance is approximated by

5 $$\begin{aligned} \tilde{\sigma }_d^2 \approx \hat{\sigma }_{v_t}^2 + \hat{\sigma }_{w_t}^2 -2\textrm{Corr}(y_{i}, \hat{\mu })\cdot \hat{\sigma }_{v_t} \cdot \hat{\sigma }_{w_t}, \end{aligned}$$

where $\textrm{Corr}(y_{i}, \hat{\mu })$ is the Pearson correlation coefficient between the WWTP estimated state time series and the observed copies/liter from the $i^{th}$ lift station. If any of the sub-sewershed values $y_{i,t}$ are missing, we replace these values with the online trend estimate for the WWTP, which will yield a value of 0.

If the sub-sewershed and the WWTP are “in control”, or gave equivalent information, then $d_{i,t}$ would be normally distributed with mean 0, and there would be no autocorrelation in the series. To determine whether the sub-sewershed is “out of control”, or separating from the trend of the WWTP, a control chart can be constructed. Many types of control charts are available for different scenarios, for example, Shewhart^27^ and cumumlative sum (CUSUM)^28^ control charts. We choose an Exponentially Weighted Moving Average control chart^29^, which can detect small shifts in temporally correlated series such as our $d_{i,t}$ and is appropriate for use with individual observations^26^. The EWMA chart is based on the following series:

6 $$\begin{aligned} z_{i,t} = \lambda d_{i,t} + (1- \lambda )z_{i,t-1}, \end{aligned}$$

where $z_{i,t}$ can be interpreted as a weighted average of all past values for series *i*, where the weighting is controlled by the value $\lambda$, for which we use the estimate of the lag 1 autocorrelation of $d_{i,t}$. In the case of a missing sub-sewershed value, the aforementioned replacement with the WWTP online estimate allows for the exponential weighting of past values to continue under the assumption of no separation. The EWMA charts are visualized for each of the 4 lift stations compared to the WWTP in Figure 3.

The dots on Fig. 3 represent the values of $z_t$. The dotted lines are the upper and lower confidence limits. When $z_t$ exceeds one of these confidence limits, the point is colored red, and the sub-sewershed can be considered “out of control”, in other words, the sub-sewershed time series is separating from the WWTP time series which gives different information. The direction of the separation can also be determined by examining whether the point exceeds the upper limit, indicating the viral concentration is higher for the sub-sewershed, or the lower limit, indicating the sub-sewershed is lower.

We summarize the creation of the EWMA chart in Algorithm 2, building off of EWMA charts and examples of their use with correlated data^30–32^.

**Algorithm 2:**
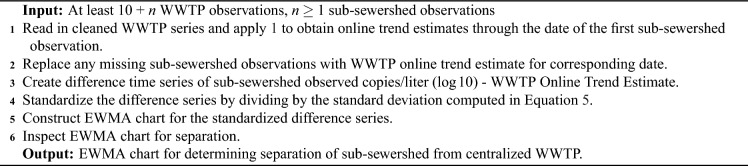
Detecting deviation of sub-sewershed measurement from centralized WWTP trend estimate.

## Conclusion

The hierarchical time series model addresses two modeling challenges important to analyzing WBE time series data: accounting for the presence of temporal autocorrelation and separating trend variability from measurement and sampling variability. Application of this hierarchical time series model provides a statistically rigorous foundation for retrospective determination of whether information collected from spatially nested sampling sites have the same trend in population disease, or whether the information gleaned differs across sampling sites. The flexible nature of this state space modeling approach can be used to add a measurement error perspective to an existing modeling approach. For example, the smoothing spline structure of the hierarchical model presented here was motivated by previous application of cubic smoothing splines^33^. This approach supports complex sampling structures such as the nested strategies thereby providing additional insight into the impact of sampling and measurement error^34^. The methodological advancements in applying the EWMA control chart provide a clear signal when information from a sub-sewershed serving a smaller population differs from that of the larger population monitored by the downstream wastewater treatment plant. This technology brings to light disease outbreaks at the community level based on wastewater measurements.

Although the methods in this paper give insight into whether the WWTP and Lift Stations give different information, as well as indicating when that difference appears, the models used here do not explicitly take spatial structure into account. Spatial models have been applied to SARS-CoV-2 wastewater viral concentration data^35^. A combination of Principal Components Analysis (PCA) and spatial autocorrelation models indicated clusters of disease hotspots, but the authors note that issues with sampling method might impact how well the data actually represent the community dynamics^36^. The state-space modeling framework can accommodate spatial structure, so the hierarchical time series model demonstrated here could be modified to a hierarchical space-time model which respects both temporal and spatial dependence structure in the data while also addressing variability due to sampling. A statistically rigorous foundation supporting WBE will better inform public health department decisions, thereby guiding prioritization of limited resources to efficiently support the community most in need, or those most burdened by disease.

## Data Availability

Given the small populations associated with some of the lift stations, real data will be made available on a case-by-case basis by contacting the corresponding author and subsequent approval by Houston Health Department. Synthetic wastewater surveillance data which preserves the statistical properties of the real data are available along with code on a GitHub repository.

## Code availibility

All code used to fit the models described in this paper are available in a public GitHub repository. All code is written in the R language^37^.
